# Obstetric haematology training: Optimising care through cultivated expertise

**DOI:** 10.1177/1753495X261426965

**Published:** 2026-02-27

**Authors:** Kristine Matusiak, Sue Pavord, Nadine Shehata

**Affiliations:** 1 Division of Hematology and Thromboembolism, Department of Medicine, 62703 McMaster University, Hamilton, Canada; 2 Department of Haematology, 6397 Oxford University Hospitals NHS Foundation Trust, Oxford, UK; 3 Departments of Medicine, Laboratory Medicine and Pathobiology, Institute of Health Policy Management and Evaluation, 7938 University of Toronto, Toronto, Canada; 4 Division of Hematology, Special Pregnancy Program, Mount Sinai Hospital, Toronto, Canada; 5 Division of Medical Oncology and Hematology, University Health Network, Toronto, Canada

**Keywords:** Pregnancy complications, hematologic

## Abstract

**Background:**

Obstetric Haematology, a subspecialty at the interface of Obstetric Medicine, Haematology and Maternal-Fetal Medicine, addresses the complex interplay between pregnancy and haematological disorders. Advances in survival and reproductive technologies have increased the number of individuals with complex haematological conditions achieving pregnancy, underscoring the need for specialised expertise. Exposure to expertise may vary significantly by training program and formal training in Obstetric Haematology remains limited.

**Methods and Results:**

A narrative review was conducted and an approach for Obstetric Hematology training is proposed consisting of a structured curriculum incorporating reproduction planning, contraception, medication safety, haematological adaptations of pregnancy, and psychosocial support.

**Conclusion:**

The development of an Obstetric Haematology curriculum can standardize education and enhance multidisciplinary collaboration with a target of improving outcomes for pregnant individuals with haematological disorders. Next steps include comprehensive needs assessment across specialties, definition of core competencies through consensus approaches, and incorporation of patient perspectives in curriculum design.

Haematological complications of pregnancy and the postpartum period, such as obstetric haemorrhage and thromboembolism, remain leading causes of maternal morbidity and mortality. Many pre-existing haematological conditions can complicate pregnancy and lead to adverse maternal and/or fetal outcomes including, but not limited to, haemoglobin disorders such as sickle cell disease, immune cytopenias, inherited and acquired thrombophilia, bleeding disorders, haematological malignancies, and red blood cell and platelet alloantibodies.^1^ Women with complex haematological disorders are achieving pregnancy due to reduced morbidity and improved survival, such as after allogeneic transplantation, and with contemporary care for individuals with sickle cell disease and beta thalassemia major.^2,3^ Furthermore, conditions such as myeloproliferative neoplasms are being identified in younger individuals.^4^ There is also increasing use of assisted reproductive technology, which often requires expert haematological consultation. Optimising outcomes for these patients requires multidisciplinary care models, also shown to be beneficial in other medical conditions such as pulmonary hypertension, cardiac disease and diabetes.^5–7^

Pregnancy is associated with several physiological changes to the blood, including expansion of red cell mass and plasma volume, leukocytosis, increased platelet activation and clearance, increased coagulation activity, and alterations in immune system function. Laboratory parameters are frequently altered during pregnancy and require expert interpretation.^8^

As such, formal training in Obstetric Haematology is necessary to address issues of reproductive care for these individuals.^2,3^ A structured curriculum and defined competencies equip Haematologists, Obstetric Medicine physicians, Maternal Fetal Medicine physicians, and Anesthesiologists with the necessary skills and knowledge to manage pregnancies and to participate in multidisciplinary discussions, to enhance the delivery of care for pregnant individuals with haematological conditions. For example, coordinated fertility programs have been shown to increase access to fertility care for those needing chemotherapy, and reduce regret about fertility preservation; yet, of 433 health care practitioners surveyed regarding the frequency of discussing fertility counselling prior to treatment, 40% indicated they never discuss fertility preservation and 56% did not feel confident in discussing these options.^9,10^

The need for Obstetric Medicine training has long been recognized, and several medical specialties have established Obstetric Medicine curricula during subspecialty and fellowship training to address this need.^11^ Needs assessments of Haematologists, Fetal Maternal Medicine specialists, Obstetricians and resident doctors, identified education in Obstetric Haematology to be highly useful.^12,13^ Haematology, General Internal Medicine, and Anesthesia training programs differ among countries in their requirements for education about the medical needs of individuals of childbearing potential. Obstetric Medicine and Maternal Fetal Medicine programs also vary in establishing specific objectives and rotations for haematology training.

Among haematology residency programs internationally, expected competencies differ (Table 1).

**Table 1.** Competencies of international haematology programs for obstetric knowledge.

Training programme	Competency
Canada^14^	Pregnancy and post-partum physiological changes and haematologic disorders
United Kingdom^15^	Alloantibodies in pregnancy, RhD disease of the newborn (including use of anti-D), neonatal alloimmune thrombocytopenia, intrauterine transfusion and transfusion in pregnancy, prevention and management of thrombosis in pregnancy, management of haematological disorders and those with a haemoglobinopathy including antenatal screening
United States The Accreditation Council for Graduate Medical Education (ACGME)^16^	Care and management of haematologic disorders in pregnant patients and patients of reproductive age; diagnosis and management of haematologic issues associated with hormone therapies, including their use as treatment for infertility

In Toronto, Canada, the Obstetric Hematology Program also provides longitudinal training to Obstetric Medicine, Maternal Fetal Medicine, Thrombosis and Haemoglobinopathy trainees. We are aware of only one fellowship program in Obstetric Haematology offered to both Haematologists and Obstetricians in Canada, and an established, multidisciplinary, three-day residential course in Oxford, UK.^17,18^ Elements for Obstetrical Haematology Training are thus suggested for Obstetric Medicine, Hematology, Obstetrics and Gynecology, Maternal Fetal Medicine and Anesthesiology training with expertise offered in a fellowship to Obstetric Medicine, Hematology and Maternal Fetal Medicine physicians.

## Considerations for an obstetric haematology curriculum

Development of an Obstetric Haematology curriculum is proposed to include disease-specific elements (Supplement, Table 1) and common elements such as approaches to:

- *Contraception counselling,* including risk assessment
- *Reproductive planning,* including impacts on fertility, sexual function, placental physiology, risk of pregnancy losses, need and access for pre-implantation or prenatal genetic diagnosis, and assisted reproductive technology.
- *Medications,* including those required to optimise disease outcomes and safe discontinuation prior to pregnancy of those with known or potential teratogenicity, characteristics of drugs that are safe for pregnancy and lactation, late effects of chemotherapy or bone marrow transplantation.
- *Cytopenias,* including the physiological changes to the blood during pregnancy, autoimmunity, recommended thresholds for intervention to support safe practices during the antenatal period, neuraxial anesthesia and childbirth.
- *Thromboprophylaxis,* including the need and duration, applying risk stratification according to the haematological disorder and other clinical and laboratory factors.
- *Risks of imaging modalities,* for diagnosis and staging haematological disease, or for investigation of suspected venous thromboembolism, potential modifications to limit radiation exposure.
- *Risks of preeclampsia and prevention*, Understanding the increased risk of hypertensive disorders of pregnancy associated with haematologic conditions and implementing risk reduction strategies.
- *Transfusion considerations,* including haematological parameters for transfusion, risks of alloimmunisation, prevention and management of obstetric haemorrhage, provision of blood components, point of care testing, and appropriate use of anti-D Immune globulin
- *Financial* assessment, for the need of costly medications, use of resources
- *Psychosocial* support

The elements suggested for inclusion are based on cases seen over 10 years in focused clinics in Canada and the United Kingdom. The incorporation of these elements will differ according to the subspecialty programme e.g., Obstetric Haematology, Maternal Fetal Medicine, and Obstetric Medicine and are advised to be selected according to a needs assessment and Delphi consensus processes. A needs assessment of obstetricians and hematology trainees in Switzerland demonstrated that the top three topics of highest interest included bleeding disorders, antiphospholipid syndrome (APS), and thrombosis in pregnancy.^13^ The Haematology elements for Obstetric Medicine have been previously identified through a similar process but could be potentially expanded with availability of training.^19^

## Fundamental educators for obstetric haematology training

**Figure 1.**
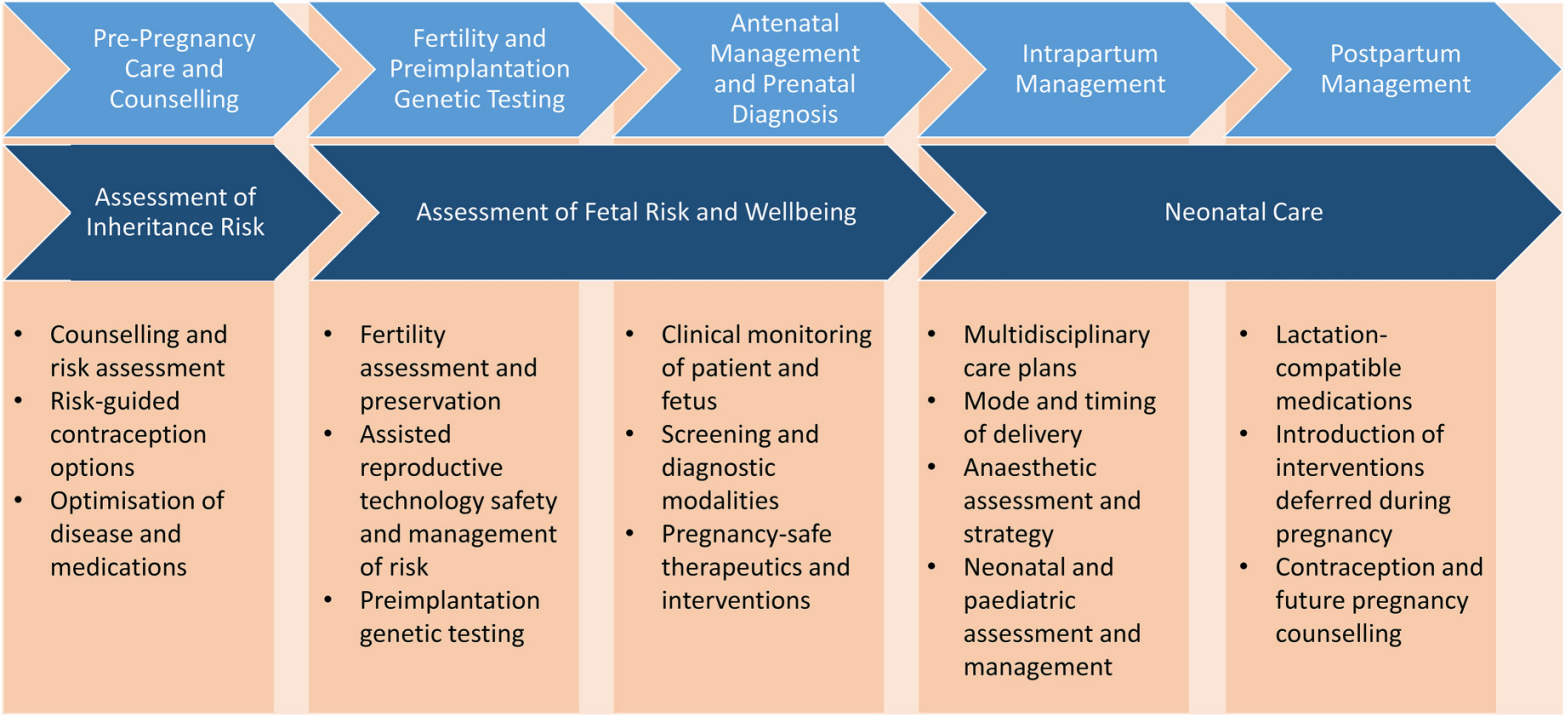
Core educational concepts across the pregnancy continuum.

As care of pregnancies with haematological disorders requires a multidisciplinary approach, an integrative style for the education of trainees is also necessary. The pathway for an individual of childbearing age may require knowledge of fertility, pregnancy and childbirth, including risks and benefits of medications and management options during this course (Figure 1). Providers for this education are proposed in Table 2.

**Table 2.** Providers for educational delivery of obstetric haematology training.

Provider specialisation	Curriculum
Obstetric Medicine	Care of comorbid conditions such as kidney disease, cardiovascular disease, endocrine disease and preventative measures for conditions such as preeclampsia
Pharmacology	Characteristics of medications that predict placental transfer (e.g., molecular weight, binding to placental Fc receptor) transfer with lactation. Adequate and safe analgesia
Reproductive endocrinology	Fertility options, assisted reproductive techniques and genetic screening
Anaesthesia	Anaesthetic modalities according to haematological and coagulation parameters
Maternal Fetal Medicine	Placental development, physiological changes, placental markers and monitoring, needs for safe birth including the postpartum period
Laboratory	Laboratory diagnosis, pregnancy-specific reference considerations (e.g., blood cell counts, transfusion medicine, platelet immunology, haemoglobin disorders)
Neonatology	Care of the newborn

## Methods and Media for curriculum development and dissemination

The clinical discipline of Obstetric Haematology has developed over the last 20–25 years, but in many countries expertise is still confined to large metropolitan academic centers.^1^ Trainee exposure to a diverse case mix and sufficient clinical volume to achieve confidence and competency may therefore be inconsistent across programs. Curriculum development with an aim to establish and maintain equitable learning opportunities for trainees must therefore be accessible, flexible and adaptable to learner settings and needs.

In a survey of haematologists, obstetricians and internists about needs and preferences for Obstetric Haematology learning, three-quarters of respondents identified a blended curriculum delivery, with combination of face-to-face teaching and technology-enhanced learning activities as their preferred mode of learning.^12^ Inclination for multidisciplinary learning with multiple specialists modelling real clinical experience was also expressed.

The use of asynchronous, self-paced modules and self-assessment provides a strategy for addressing disparate trainee experiences due to limited clinical exposure or local content expertise.^13^ As an example, the care of individuals with haemoglobinopathies has limited exposure in Canada.^20–22^ Similarly, there has been a paucity of educators in transfusion medicine and immunohaematology, leading to inconsistent trainee exposure.^23^ The development and use of online modules to provide foundation training in clinical and laboratory content was found to be an acceptable method and was rated favourably in improving understanding and examination scores in a Canadian setting.^24^ Knowledge scores of Haematology trainees improved in antibody panel interpretation, an area previously identified as a weakness.^23,24^ This approach was also found to be acceptable for Obstetric Hematology training, and can be made available on educational websites.^13^

A blended didactic and case-based learning style is valued, as undertaken in the international course held in Oxford, U.K.^17^ It has also been developed for Transfusion Medicine education for trainees from multiple specialties. The development and implementation of Transfusion Camp designed for Haematology, Anaesthesia, Critical Care, and other subspecialty trainees, has been demonstrated to improve trainee knowledge and attitudes as well as self-reported practice implementation about fundamental transfusion medicine topics.^25,26^ These findings have been replicated in the United Kingdom.^27^ A blended learning program could also be applied to an Obstetric Haematology curriculum. Using the above curriculum framework, a combination of self-paced online content and interactive case-based learning sessions could be devised and implemented for Haematology trainees.

## Conclusion and next steps

The need for Obstetric Haematology training is evident. This is a critical area of medicine which requires high-level multidisciplinary expertise to optimise outcomes. Next steps should include a comprehensive needs assessment among the various subspecialty disciplines, engagement of other providers needed for educational delivery, and a Delphi approach to determine core competencies. Incorporating the patient perspective is also crucial for curriculum design.

## Supplemental Material

sj-docx-1-obm-10.1177_1753495X261426965 - Supplemental material for Obstetric haematology training: Optimising care through cultivated expertise Supplemental material, sj-docx-1-obm-10.1177_1753495X261426965 for Obstetric haematology training: Optimising care through cultivated expertise by Kristine Matusiak, Sue Pavord and Nadine Shehata in Obstetric Medicine

## References

[1] Pavord S Oppenheimer C . Reflections on the development of obstetric haematology as a new discipline in the UK. Br J Haematol 2020; 191: 604–607. 33190262 10.1111/bjh.17165

[2] Quinn CT Rogers ZR McCavit TL , et al. Improved survival of children and adolescents with sickle cell disease. Blood 2010; 115: 3447–3452. 20194891 10.1182/blood-2009-07-233700 PMC2867259

[3] Sockel K Neu A Goeckenjan M , et al. Hope for motherhood: pregnancy after allogeneic hematopoietic cell transplantation (a national multicenter study). Blood 2024; 144: 1532–1542. 39007722 10.1182/blood.2024024342

[4] Robinson S Ragheb M Harrison C . How I treat myeloproliferative neoplasms in pregnancy. Blood 2024; 143: 777–785. 38145575 10.1182/blood.2023020729

[5] Chen W Luo J Chen J , et al. Effect of multidisciplinary team (MDT) centred on pregnant women with pulmonary hypertension on treatment and outcomes of pregnancy. BMC Pulm Med 2023; 23: 62. 36765334 10.1186/s12890-023-02355-1 PMC9921663

[6] Li H Li H Wang J , et al. Effectiveness of multidisciplinary intervention for gestational diabetes mellitus. J Obstet Gynaecol Res 2023; 49: 863–869. 36697857 10.1111/jog.15519

[7] Magun E DeFilippis EM Noble S , et al. Cardiovascular care for pregnant women with cardiovascular disease. JACC 2020; 76: 2102–2113. 33121718 10.1016/j.jacc.2020.08.071

[8] Paidas MJ Hossain N . Hematologic changes in pregnancy. In: Hemostasis and thrombosis in obstetrics & gynecology. Chichester, West Sussex, UK: Wiley-Blackwell, 2010, pp.1–11.

[9] Letourneau JM Ebbel EE Katz PP , et al. Pretreatment fertility counseling and fertility preservation improve quality of life in reproductive age women with cancer. Cancer 2012; 118: 1710–1717. 21887678 10.1002/cncr.26459 PMC3235264

[10] Lopategui DM Ibrahim E Aballa TC , et al. Effect of a formal oncofertility program on fertility preservation rates-first year experience. Transl Androl Urol 2018; 7: S271–S275. 10.21037/tau.2018.04.24 PMC6087850 30159232

[11] Cumyn A Gandhi S Gibson P , et al. Defining competencies for training in obstetric medicine. Obstet Med 2014; 7: 137–138. 27512441 10.1177/1753495X14551244 PMC4934984

[12] Kazi S Tseng E Malinowski A , et al. A survey to assess the educational needs of clinicians in maternal hematology. Blood 2020; 136: 9–10.

[13] Sutter T Vandermeulen H Shehata N , et al. Targeted online education in obstetric hematology significantly improves resident knowledge and addresses a critical training gap. Blood 2025; 146: 4640–4640.

[14] Royal College of Physicians and Surgeons of Canada. Hematology Competencies (accessed July 2025).

[15] The Joint Royal College of Physicians Training Board. Haematology Training Curriculum Implementation. 2021.

[16] ACGME Program Requirements for Graduate Medical Education in Hematology and Medical Oncology. Available at https://www.acgme.org/globalassets/pfassets/programrequirements/2025-reformatted-requirements/155_hematologymedicaloncology_2025_reformatted.pdf (accessed December 2025).

[17] British Society for Haematology. Haematology in Obstetrics course. 2015.

[18] University of Toronto. Maternal Fetal Hematology [Available from: https://deptmedicine.utoronto.ca/maternal-fetal-hematology#:∼:text=This%20fellowship%20in%20Maternal%2FFetal,with%20hematological%20disorders%20in%20pregnancy.

[19] Cumyn A Gibson P . Validation of a Canadian curriculum in obstetric medicine. Obstet Med 2010; 3: 145–151. 27579080 10.1258/om.2010.100038 PMC4989631

[20] Bhella S Radwi M Ward R . An online educational module to improve general internal medicine Trainees’ knowledge and comfort in managing acute complications of sickle cell disease. J Appl Hematol 2023; 14: 242–247.

[21] Verhovsek MM Breakey VR Ward R , et al. Hemoglobinopathy education in Canadian hematology training programs: how much are residents learning? Blood 2014; 124: 2168–2168.25278567

[22] Verhovsek MM Hou C Azzam M , et al. Pilot study of online learning modules for hemoglobinopathy education in Canadian hematology training programs. Blood 2016; 128: 314.28092879

[23] Yan MTS Arsenault V Pendergrast J . Evaluation of immunohematology knowledge in hematology trainees. Transfusion 2019; 59: 2685–2690. 31150568 10.1111/trf.15390

[24] Arsenault V Yan MTS Tait G , et al. An online immunohematology educational resource for post-graduate hematology trainees: learnSerology.ca. Transfus Apher Sci 2023; 62: 103634. 36566086 10.1016/j.transci.2022.103634

[25] Lin Y Tilokee E Charge S , et al. Transfusion camp: a prospective evaluation of a transfusion education program for multispecialty postgraduate trainees. Transfusion 2019; 59: 2141–2149. 30946497 10.1111/trf.15284

[26] Yeung KCY Kapitany C Charge S , et al. Transfusion camp: a retrospective study of self-reported impact on postgraduate trainee transfusion practice. Transfusion 2023; 63: 839–848. 36811164 10.1111/trf.17278

[27] Aggarwal A Kaushik K Morton S , et al. Transfusion camp: the UK experience and its value in improving knowledge of transfusion medicine among postgraduate trainees. Transfus Med 2024; 34: 450–454. 39117599 10.1111/tme.13075

