# Supplementary material for: Obstetric haematology training: Optimising care through cultivated expertise
Source: Obstet Med. 2026 Feb 27:1753495X261426965. Online ahead of print. doi: 10.1177/1753495X261426965 (PMC12949731; doi:10.1177/1753495X261426965)
Supplement: sj-docx-1-obm-10.1177_1753495X261426965 - Supplemental material for Obstetric haematology training: Optimising care through cultivated expertise [file sj-docx-1-obm-10.1177_1753495X261426965.docx]

Supplementary Table 1. Proposed Obstetric Haematology Curriculum

| Disease | Educational objectives |
| --- | --- |
| Hematologic Malignancies | |
| Acute Hematologic Malignancy – Leukemia and Lymphoma and Hematopoietic Stem Cell Transplant | Fertility counselling and preservation pre-pregnancy and at birth  Options, risks, and timing of chemotherapy during pregnancy and when anticipating pregnancy  Use and safety of supportive care options (e.g. anti-emetics, antibiotics, anti-virals, anti-emetics)  Safety of diagnostic procedures (bone marrows, lymph node biopsies) and radiological imaging during pregnancy  Transfusion thresholds antepartum, at birth and postpartum  Timing of transplantation |
| Chronic myeloid leukemia (CML) | Fertility counselling  Risks of CML in pregnancy  Criteria/risks and benefits for discontinuing or pausing Tyrosine Kinase Inhibitor (TKI) therapy  TKI options during pregnancy  Options for disease control during pregnancy and lactation |
| Philadelphia negative myeloproliferative neoplasms (MPN) | Risk stratification for pregnancy including thrombotic and hemorrhagic risks and risks according to molecular mutations/allele burden  Pregnancy complications secondary to MPNs  Options and risks of treatment during pregnancy and postpartum |
| Bone marrow failure syndromes | Transfusion thresholds antepartum, at birth and postpartum  Use of growth factors during pregnancy and lactation  Options for treatment/supportive care |
| Inherited Red Cell Disorders | |
| Sickle cell disease | Maternal and fetal complications of sickle cell disease  Genetic counselling and preimplantation genetic diagnosis  Assessment of fertility and pregnancy loss  Pre-pregnancy optimization including iron homeostasis, organ function, discontinuation of medication, vaccination  Indications for red cell on demand and exchange transfusion and optimizing red cell products (i.e. phenotyping, genotyping, providing matched red blood cells)  Use of medications including opioids, NSAIDs, ASA, hydroxyurea, iron chelation during pregnancy and lactation  Need and use of anticoagulation  Optimizing mode of delivery  Timing and implications of transplantation, gene therapy |
| Thalassemia major | Maternal and fetal risks of thalassemia major  Genetic counselling and preimplantation genetic diagnosis  Effect of iron overload on fertility  Iron homeostasis prior and during pregnancy  Use of iron chelation during pregnancy and lactation  Transfusion needs and provision of phenotyped/genotyped red cells |
| Other congenital red cell disorders | Genetic counselling  Transfusion criteria  Pre-pregnancy optimization including splenectomy |
| Cytopenias | |
| Hematinic deficiencies (e.g. iron, B12) | Pre-pregnancy optimization  Physiologic changes in hematological parameters and hematinic levels during pregnancy  Pregnancy- and fetal/neonatal risks of anemia and hematinic deficiencies  Diagnostic thresholds for anemia and nutritional deficiencies in pregnancy  Approach to hematinic replacement in pregnancy |
| Immune thrombocytopenia (ITP) | Pre-pregnancy optimization  Indications for treatment, the use of first and second line agents for ITP and maternal and fetal adverse effects of these agents  Platelet thresholds for neuraxial anesthesia and vaginal and Caesarean deliveries  Risk of neonatal thrombocytopenia |
| Autoimmune hemolytic anemia | Pre-pregnancy optimization  Fetal risks of AIHA  Indications for treatment, the use of first and second line agents and maternal and fetal adverse effects of these agents  Transfusion support |
| Microangiopathies | |
| Congenital/acquired microangiopathic hemolytic anemias (TTP/aHUS) | Pre-pregnancy optimization  Criteria for diagnosis during pregnancy, treatment options, risk of relapse in subsequent pregnancies and indications for treatment, use of anti-platelet medications and anticoagulants  Pregnancy considerations for apheresis treatment  Genetic screening for congenital disorders |
| Hypertensive disorders of pregnancy | Preeclampsia and Hemolysis Elevated Liver enzymes Low Platelet (HELLP) assessment and hematologic management |
| Acute fatty liver of pregnancy | Assessment and hematologic management |
| Hemorrhagic Disorders | |
| Bleeding Assessment | Bleeding assessment tool criteria, criteria for safe neuraxial anesthesia and birth  Appreciate changes in coagulation parameters during pregnancy and postpartum  Appreciate limitations in diagnostic evaluation of vWD, platelet function defects, and the use of coagulation parameters peripartum |
| Von Willebrand disease | Pre-conception counseling  Laboratory testing during pregnancy  Genetic counseling  Management of bleeding risk for invasive procedures, neuraxial anesthesia and delivery  Criteria for neonatal diagnosis |
| Hereditary coagulation factor deficiencies | Pre-conception counseling  Laboratory testing during pregnancy  Genetic counseling and prenatal testing  Management of bleeding risk for invasive procedures, neuraxial anesthesia and delivery  Criteria for neonatal diagnosis |
| Congenital platelet function defects | Pre-conception counseling  Laboratory testing during pregnancy  Genetic counseling  Management of bleeding, risk for invasive procedures during pregnancy, neuraxial anesthesia and delivery  Use of transfusion and risks of alloimmunization |
| Acquired coagulation deficiencies | Use of immunosuppression during pregnancy  Use of bypassing agemts in pregnancy  Management of bleeding risk for invasive procedures, neuraxial anesthesia and delivery |
| Obstetric hemorrhage and DIC | Prevention and optimization for patients at increased risk of obstetric hemorrhage  Acute management strategies for obstetric hemorrhage, including use of anti-fibrinolytics and blood products |
| Thromboembolism and Anticoagulation | |
| Thrombotic disorders | Hemostatic changes in pregnancy that result in a thrombophilic predisposition and the duration of thrombophilic risk  Risk stratification for anticoagulation for patients with   1. mechanical heart valves 2. congenital heart disease 3. hereditary thrombophilias 4. prior venous thromboembolism 5. prior arterial thromboembolism, including stroke   Risks and accuracy of diagnostic investigations ( chest x-ray, ultrasonography, CT, MRI)  Diagnostic strategies for VTE  Prophylaxis and therapeutic anticoagulation needs and dosing during fertility, pregnancy and lactation for high-risk disorders including anti-phospholipid syndrome and mechanical heart valves  Need for screening for hereditary and acquired disorders  Duration of anticoagulation  Contraception counselling for patients at increased risk of thrombosis |
| Antiphospholipid Syndrome (APS) | Laboratory diagnosis of APS  Obstetric diagnosis for APS  Fertility options for APS  Antepartum and postpartum management: anticoagulation, hydroxychloroquine, complement-directed therapies |
| Recurrent Pregnancy Loss | Indications and limitations of investigation and management from thrombosis/hemostasis perspective of   1. Recurrent 1st pregnancy loss 2. 2nd or 3 trimester loss 3. Primary and secondary infertility |
| Fetal Maternal Alloimmune Syndromes | |
| Hemolytic Disease of the Fetus and Newborn (HDFN) | Risk of development of HDFN according to alloantibody  Surveillance of at risk pregnancies  Use of non-invasive prenatal testing for fetal diagnosis  Prevention of alloimmunization  Management of fetal anemia  Optimization prior to delivery and sourcing of rare red blood cells, where applicable |
| Fetal/Neonatal Alloimmune Thrombocytopenia (FNAIT) | Pathophysiology of FNAIT  Laboratory investigation for FNAIT  Options and limitations for invasive and non-invasive treatment antenatally and for postnatal management |

aHUS, atypical hemolytic uremic syndrome, AIHA, autoimmune hemolytic anemia, APS, Antiphospholipid Syndrome, CML, chronic myeloid leukemia, FNAIT, fetal neonatal alloimmune thrombocytopenia, HDFN, hemolytic disease of the fetus and newborn, ITP, immune thrombocytopenia, MPN, myeloproliferative neoplasm, NSAIDs, non-steroidal anti-inflammatory medication, TTP, thrombotic thrombocytopenic purpura, VTE, venous thromboembolism
